# 

**Published:** 1895-10-12

**Authors:** 


**Notices of Births, Deaths, and Marriages** are inserted in this column at a charge of 2s. 6d. for an announcement not exceeding four lines.

**Notice to Advertisers and Subscribers.**

All Advertisements, Orders for Copies of The Hospital and Business Communications should be addressed to **THE MANAGER, (Not to The Editor)**, The Hospital, 428, Strand, London, W.C.

The Cost of Subscription, post free, is as follows:—Per week, 2½d.; per quarter, 2s. 8d.; per half-year, 5s. 3d.; per annum, 10s. 6d. Foreign:—Per Annum, 15s. 2d.; payable, in all cases, in advance.

### NOTICE TO CORRESPONDENTS.

All MS., Letters, Books for Review, and other matters intended for the Editor should be addressed The Editor, The Lodge, Porchester Square, London, W.

The Editor cannot undertake to return rejected MS., even when accompanied by stamped directed envelope.

**"Burdett's Hospital Annual," 1895.**

Sixth year of publication. 950 pp. Scarlet cloth, gilt lettered. Price 5s. A most complete guide to British, Colonial, Indian, and American Hospitals, Dispensaries, Nursing and Convalescent Institutions, and Asylums. A few complete sets of the preceding five issues of the "Annual" may still be obtained on application to the Publishers, The Scientific Press (Limited), 428, Strand, London, W.C.

**NOTICE.—Vol. XVIII. of "The Hospital" (April to September, 1895), in handsome cloth binding, is now on sale at the Office of this Journal. Price 6s. Cases for binding the Numbers contained in this Volume, 1s. 6d. Index to Vol. XVIII., 2½d., post free.**

**The Monthly Part for October is now ready, price 6d.; by post, 8d.**

### Scraps and Gleanings.

The sum realised by the Hospital Saturday Collection at Belfast was £300.

The Nursing Institute, in connection with the Merthyr General Hospital, is now complete.

The Sunderland Eye Infirmary relieves over 1,000 persons suffering from disease and accident to the eye each year.

The Mayor and Corporation of Eastbourne have invited the members of the British Medical Association to meet there next year.

The receipts of the Devizes Cottage Hospital show a satisfactory increase, and include a legacy of £100 from the late Mr. Biggs.

Over 50,000 persons visited the temporary mortuary chapel at the Pasteur Institute and defiled before the coffin of the late savant.

The city physician and coroner at Pender is a woman, who received the unanimous vote of the citizens after a period of six months' practice there.

The German Emperor has promised a handsome subscription towards the proposed Helmholtz Statue at Berlin; this subscription will amount to £500.

At the British Medical Association no less than 82 physicians from Canada and the United States were numbered among the guests and visitors.

The sum of £500 has yet to be forthcoming in discharge of the amount due on the new buildings of the Alloa New County Fever Hospital.

The rainfall of last week was in excess of the average over nearly all the United Kingdom. The total amount in London was more than double the normal.

The amount of this year's Hospital Sunday collection at Hull has been augmented to the extent of £18 by a generous donor, who deposited that amount to the credit of the fund.

There are about 12 hospitals in China and 25 medical men professionally educated, a small enough number when we bear in mind that the Celestial Empire numbers 400,000,000 in population.

The income of Market Rasen Dispensary and Cottage Hospital has lately not been sufficient to meet the yearly expenses, and in their annual report the committee make an appeal for additional help.

The Local Government Board have authorised the expenditure, and approved of the loan of an additional £10,000 in connection with the erection of a new administrative block at the North-Western Hospital.

Professor Bunge read a paper at the German Congress of Medicine, drawing attention to the need of increasing our attention to the consumption of food containing iron as a substitute for its administration in drugs.

The Westminster Hospital, which has been closed for repairs for nearly two months, opened last week for out-patients. During the interval the building has been thoroughly overhauled, and some important sanitary improvements effected.

M. Pasteur has left a will in which he simply says: "I leave to my wife all that the law permits me to leave her. May my children never neglect the law of duty ever to preserve towards her, their mother, the feeling of tenderness which she deserves."

The premises of the Victoria Dental Hospital at Manchester having proved themselves inadequate to the increasing calls upon their resources, a new site, we learn, has been bought, which is at present lying idle, no money being available for building purposes.

The fifteenth report of the inspector of retreats under the Inebriates Acts has been issued for 1894. Nine such retreats are licensed under these Inebriates Acts. There is a satisfactory report of the successful nature of the scheme; there have been 133 admissions during the twelve months.

The plans of the Isle of Thanet Infectious Diseases Hospital have met with the approval of the Local Government Board after certain additions, which will necessarily increase the cost. To meet these the seal of the Board was fixed to the issue of certain debentures to secure a loan of £2,000.

The autumn half-yearly meeting of the Bolton Working Men's Hospital Saturday Fund shows an encouraging result, these collections now having turned their second thousand pounds; and this advance was made in face of a depressed state of the cotton trade, which had suffered more than in many years past.

The anniversary service in connection with the Salisbury Infirmary has taken place at the Cathedral, the Mayor and members of the Corporation, infirmary governors, and patients walking in procession to the place of worship. Over £32 were thus collected towards the finances of the institution.

The conference recently held at Durham of members of Urban and District Councils to discuss the question of providing isolation hospitals throughout the county is calculated to serve a very useful purpose. The towns are well provided for, but there is a pressing need for such accommodation in almost every rural district in the county.

The thirteenth annual report of the Yorkshire Home for Chronic and Incurable Diseases, at Harrogate, shows that the institution is doing a good work among sufferers reduced in circumstances through long-continued affliction. The balance-sheet has a small sum in favour of the funds, but unfortunately there are against this certain outstanding debts.

The forty-fifth report of the Commissioners in Lunacy shows that their number has increased since last year's statistics were published. It has been pointed out that this may be readily accounted for by the fact that a great many patients are received in asylums at the present time from causes of mental decay through old age, who previously found admittance harder.

A Local Government inquiry was held the end of last month at Cardiff, in reference to the application from the town to erect a cholera hospital on Flat Holm Island, in the British Channel. The existing building is little more than a shed, capable of holding six beds. Barry joins with Cardiff in this proposed erection, the expense of hospital provision to be borne by each authority *pro rata* to the tonnage entering each place.

### The Student of Medicine.

### APPOINTMENTS.

C. C. Aitken, M.B., C.M.Edin., appointed Junior House Surgeon to Cumberland Infirmary, Carlisle. P. T. B. Beale, F.R.C.S.Eng., appointed Demonstrator of Histology and reappointed Lecturer on Elementary Biology in King's College, London. J. Biernacki, M.B., C.M.Glasg., L.R.C.P.Edin., appointed Medical Superintendent of the West Ham Fever Hospital. A. E. Boulton, M.R.C.S.Eng., L.S.A., appointed Medical Officer of Health to the Horncastle Rural District Council. J. G. E. Colby, M.A.Oxon, M.B., B.Ch., appointed Medical Officer of Health for the Malton Rural Sanitary District and for the Morton Rural Sanitary District. A. B. Collins, L.R.C.P.Lond., M.R.C.S., appointed Medical Officer for the Yapton District of the Westhampnett Union. Mr. Cook, appointed Assistant Medical Officer to the Poorhouse of the Greenock Parish Council. W. K. Fyffe, M.B., M.R.C.P., appointed Assistant Physician to the City of London Hospital for Diseases of the Chest, Victoria Park. G. G. Gidley, L.R.C.P.Lond., M.R.C.S., appointed District Medical Officer to the Tiverton Union. J. Gordon-Smith, L.R.C.P., and L.R.C.S.Edin., appointed Assistant Colonial Surgeon, Gold Coast. F. W. P. Holton, M.R.C.S.Eng., L.S.A., appointed Medical Officer for the Tenth District of the West Ham Union. Mr. J. W. Johnston, appointed Medical Officer for the Workhouse and First District of the Fordingbridge Union. B. C. Kendall, M.R.C.S., L.R.C.P.Lond., appointed Medical Officer for the Wendron District of the Helston Union. Dr. F. Laird, appointed Medical Officer for the Raventhorpe District of the Dewsbury Union. T. C. Lawson, M.R.C.S Eng., L.S.A., appointed Medical Officer for the Stogursey District of the Williton Union. D. A. Leigh, appointed Medical Officer for the Malpas District of the Whitchurch Union, Salop. J. Macdonald, M.A.Edin., M.B., appointed Medical Officer of Health to the Carlisle Rural District Council. J. McNaught, M.D., appointed Medical Officer of Health to the Rawtenstall Town Council. H. G. H. Monk, M.R.C.S., D.P.H.Eng., appointed Medical Officer of Health and Public Analyst to the Leicester Town Council. Mr. W. H. Richards, appointed District Medical Officer to the Cardiff Union. J. A. Robertson, M.D.Glasg., appointed Medical Officer of Health for Peterborough. Mr. F. Romer, appointed Medical Officer for the No. 3 District of the Malmesbury Union. Dr. J. T. Thomas, appointed Medical Officer of Health and Port Medical Officer at Lowestoft. T. H. L. Tylecote, appointed House Surgeon to the Lancaster Infirmary. Dr. A. Walker, appointed Medical Officer of Health to the Weetslade Urban District Council. Dr. G. H. Weston, appointed Medical Officer for the No. 3 District of the South Stoneham Union.

### VACANCIES.

The following vacancies are announced this week, the amount of salary in each case being given after the name of the institution:—

#### Resident Medical Officers.

Chesterfield and North Derbyshire Hospital and Dispensary, Chesterfield.—Junior House Surgeon and Dispenser. Salary £50 per annum, with board, apartments, and laundress. Applications to the Secretary by October 17th.

Essex and Colchester Hospital.—House Surgeon, doubly qualified. Salary £80 per annum, with board and lodging in the hospital. Applications to the Committee by October 18th.

Manchester Hospital for Consumption and Diseases of the Chest.—Resident Medical Officer for the Institution at Bowdon, Cheshire. Salary £60 per annum, with board, apartments, and washing. Applications to C. W. Hunt, Secretary, by October 14th.

Royal Free Hospital, Gray's Inn Road, W.C.—Resident Medical Officer (House Physician), doubly qualified. Appointment for six months, but eligible for re-election. Board, residence, and washing provided. No salary. Applications to the Secretary by October 19th.

Royal Hospital for Diseases of the Chest, City Road; E.C.—Resident Medical Officer. Appointment for six months, when re-election is required. Salary at the rate of £100 per annum, with furnished apartments and board. Applications to the Secretary by October 15th.

South Devon and East Cornwall Hospital, Plymouth.—House Surgeon. Salary £100, with board and residence. Applications and testimonials to J. Walter Wilson, Honorary Secretary, by October 12th.

South Devon and East Cornwall Hospital, Plymouth. — Assistant House Surgeon. Appointment for six months, but renewable for a further period of six months. Board and residence and an honorarium of £10. Applications to J. Walter Wilson, Honorary Secretary, by October 19th.

Sussex County Hospital, Brighton.—Fourth Resident Medical Officer, doubly qualified, unmarried, and under 30 years of age. Salary not exceeding £30 per annum, with board, washing, and residence in the hospital. Applications to the Secretary by October 23rd.

#### Non-Resident Medical Officers.

London Hospital, Whitechapel, E.—Medical Electrician; must be qualified and registered under the Medical Act. Applications to G. Q. Roberts, House Governor, by October 19th.

### THE STUDENT OF MEDICINE—*continued.*

Carnarvon Joint Sanitary District.—Medical Officer of Health, must be between 25 and 40 years of age, doubly qualified. Must devote his whole time to the duties, and have a knowledge of the Welsh language. Appointment for five years. Salary £694 per annum, inclusive of all expenses, except those incurred for such books, stationery, and apparatus required in the performance of the duties. Applications, endorsed "Application for Office of M. O. Health," to J. H. Thomas, Clerk to the Joint Committee, 14, Market Street, Carnarvon, by October 16th.

East London Hospital for Children and Dispensary for Women, Glamis Road, Shadwell, E.—Assistant Physician to see out-patients. Must be a Fellow or Member of the Royal College of Physicians of London. Applications to Thomas Hayes, Secretary, by October 26th.

East Sussex County Council.—Consulting Medical Officer. Appointment for one year. Honorarium, 100 guineas and travelling expenses Applications to F. Ferrifield, Clerk to the County Council, County Hall, Lewes, by October 14th.

Glasgow Maternity Hospital.—Obstetric Physician and Assistant Obstetric Physician. Applications to Arthur Forbes, Secretary, 146, Buchanan Street, Glasgow, by November 8th.

### UNIVERSITIES AND COLLEGES.

**University of Durham.**

Second Examination for the Degree of Bachelor in Medicine.

The following candidates have satisfied the examiners:—

Anatomy, Physiology, Materia Medica.—First Class Honours: G. G. Turner, College of Medicine, Newcastle-upon-Tyne; E. G. E. Arnold, M.R.C.S., L.R.C.P., St. Thomas's Hospital. Second Class Honours: V. Burrow, St. Mary's Hospital; R. F. Moorshead, Bristol Medical School; T. Streatfield, M.R.C.S., L.R.C.P., University College Hospital, London; H. C. Coxon, College of Medicine, Newcastle-upon-Tyne. Pass List: J. E. Baker, College of Medicine, Newcastle-upon-Tyne; F. A. Cooke, College of Medicine, Newcastle-upon-Tyne; W. W. Deans, Yorkshire College, Leeds; C. E. Fenn, King's College; J. M. Gover, College of Medicine, Newcastle-upon-Tyne; C. Johnson, College of Medicine, Newcastle-upon-Tyne; R. A. R. Lankester, University College Hospital, London; W. H. Lister, College of Medicine, Newcastle-upon-Tyne; W. B. Milbanke, College of Medicine, Newcastle-upon-Tyne; G. W. Middlemiss, College of Medicine, Newcastle-upon-Tyne; F. Pope, Mason College, Birmingham; H. C. Regnart, Guy's Hospital; B. C. Stevens, St. Thomas's Hospital.

**Guy's Hospital.**

Entrance Scholarships.

An Entrance Scholarship in Arts of the value of £100 was awarded to Mr. G. Lewin, of 26, Orlando Road, Clapham, S.W., who was educated at the City of London School; and the Second Entrance Scholarship in Arts, of the value of £50, was awarded to Mr. F. G. Gibson, of 145, Shooter's Hill Road, Blackheath, S.E., who was educated at Canterbury College, New Zealand. Certificates of Honour were obtained by the following: Mr. M. Feldman, Jews' College; Mr. F. S. S. George, Dulwich College; Mr. H. F. Hatfield, Dulwich College; and Mr. H. D. Kempthorne, St. John's Foundation School, Leatherhead.

The Entrance Scholarship in Arts of the value of £30 for Dental Students was awarded to Mr. H. A. G. Butler, Douglas School, Cheltenham.

The Entrance Scholarship in Science, of the value of £150, was awarded to Mr. P. W. L. Camps, of 25, Cross Street, Islington, N.; and the Second Entrance Scholarship in Science, of the value of £60, was awarded to Mr. S. Hodgson, of 201, Brixton Hill, S.W. Certificates were awarded to Mr. E. A. Miller and Mr. D. Forsyth.

**University College, London (Session 1895-96.)**

Medical Entrance Exhibitions.—131 Guineas, T. C. Savage; 55 guineas, H. W. Reynolds; and 55 guineas, W. R. Scroggie.

**Account Books for Institutions; Designed in accordance with the Uniform System of Accounts for Hospitals and Institutions.**

By HENRY C. BURDETT.

Being a complete set of Royal, Foolscap, and Double Foolscap Account Books, ruled in accordance with the Uniform System adopted by the Metropolitan Hospital Sunday Fund. Designed and constructed for the convenience and assistance of Secretaries who present annual returns for participation in the Hospital Sunday Fund Grants.

Descriptive Price List post free on application.

Demy 8vo., profusely Illustrated with Specimen Tables, &c. Price 6s., post free.

**The Uniform System of Accounts, Audits, and Tenders for Hospitals and Institutions.**

Being a Key to the Method of Keeping Account Books for Institutions as above.

London: The Scientific Press (Limited), 428, Strand, W.C.

Ready October 15th. With numerous Illustrations, demy 8vo., blue buckram, gilt, 3s. 6d.

### DIET IN SICKNESS AND IN HEALTH.

**By Mrs. ERNEST HART,**

*Formerly Student of the Faculty of Medicine of Paris, and of the London School of Medicine for Women.*

With an Introduction by Sir HENRY THOMPSON, F.R.C.S., M.B. Lond.

A Practical Treatise on Diet both in Sickness and Health, based upon the most recent scientific and medical knowledge; but written in Popular and Comprehensive Language, and accompanied by a Series of Valuable Recipes for the Feeding of Invalids and others.

Sir Henry Thompson, in his Introduction, says:—"I do not hesitate to express my opinion that the present volume forms a handbook to the subject, which will not only interest the dietetic student, but offer him, within its modest compass, a more complete epitome thereof than any work which has yet come under my notice."

London: THE SCIENTIFIC PRESS (LIMITED), 428, STRAND, W.C.

Just Published. Crown 8vo., 6s.

### PIONEER WORK IN OPENING THE MEDICAL PROFESSION TO WOMEN:

**Autobiographical Sketches.**

By DR. ELIZABETH BLACKWELL.

London: Longmans, Green, and Co.

### NURSING BOOKS AT FULL DISCOUNT.

*LIST POST FREE.*

The Theory and Practice of Nursing (Percy Lewis, M.D.), 3/6 for **2/8.**

The Nurse's Dictionary (Honnor Morten), 2/- for **1/6.**

The Nurse's Case Book, 6d. for **3½d.**

### ROYAL NATIONAL PENSION FUND FOR NURSES.

*President*—**H.R.H. THE PRINCESS OF WALES.**

*Chairman*—WALTER H. BURNS, Esq. *Deputy-Chairman*—HENRY C. BURDETT, Esq.

**PENSIONS. SICKNESS. ACCIDENT.**

### Total Invested Funds, £200,000.

Nurses are invited to join the Fund on account of the substantial and exceptional advantages which it offers them and which *they cannot obtain elsewhere.* The following are the chief points :—

1. **The Fund is Mutual and essentially Co-operative.**
2. **Easy Payment of Premiums.**
   Nurses can pay their premiums monthly or otherwise as best suits their convenience.
3. **The Fund is open to every Nurse.**
   Nurses can assure for Pensions of any amount commencing at any age.
4. **An Investment and Savings Bank.**
   Those entering under the returnable scale can have their premiums returned to them with compound interest, less a small deduction for working expenses. Interest allowed on deposits.
5. **Additions to Pensions.**
   Besides the Pension entered for, substantial additions thereto may be anticipated in the shape of bonuses.
6. **Sickness and Accident Assurance.**
   Policies are issued in connection with Pension policies assuring 10s., 15s., or 20s. a week in cases of incapacity from work through sickness or accident.

**Full particulars to be obtained from THE SECRETARY,**

ROYAL NATIONAL PENSION FUND FOR NURSES,

28, FINSBURY PAVEMENT, LONDON, E.C

### STANDARD TEXT-BOOKS FOR NURSES.

New and Revised Edition. Demy 8vo., 209 pp., profusely illustrated with Copyright Portraits and Drawings. Price 2s. 6d., post free.

HOW TO BECOME A NURSE : And How to Succeed. A complete guide to the Nursing Profession for those who wish to become Nurses, and a useful book of Reference for Nurses who have completed their training and seek employment. Compiled by HONNOR MORTEN (Author of "Sketches of Hospital Life," "The Nurse's Dictionary," &c.)

"To those who are frequently appealed to by girls or youug women, as to the steps they should take to become nurses, this book of Miss Morten's must prove a perfect godsend."—*British Medical Journal.*

THE NURSES' DICTIONARY OF MEDICAL TERMS AND NURSING TREATMENT. By HONNOR MORTEN. 2nd Edition. Demy 16mo. Cloth 2s., leather 2s. 6d.

NURSING. By ISABEL ADAMS HAMPTON. 2nd Edition. Crown 8vo. Cloth, 500 pp. 7s. 6d. net.

ART OF MASSAGE. By A. CREIGHTON HALE. Demy 8vo. Cloth. Illustrated. 6s.

SURGICAL WARD-WORK AND NURSING. By ALEXANDER MILES, M.D. (Edin.), C.M., F.R.C.S.E. Demy 8vo. Cloth. With numerous illustrations. 3s. 6d.

HOSPITAL SISTERS AND THEIR DUTIES. By EVA C. E. LUCKES. 3rd Edition. Crown 8vo. Cloth. 2s. 6d.

ART OF FEEDING THE INVALID. By a Medical Practitioner and a Lady Professor of Cookery. Demy 8vo. Cloth, 260 pp. 3s. 6d.

London: The Scientific Press (Ltd.), 428, Strand, W.C.

